# Accommodative responses under various viewing conditions in surgical patients with intermittent exotropia: an institutional, retrospective study

**DOI:** 10.1186/s12886-022-02434-7

**Published:** 2022-05-04

**Authors:** Ziyi Qi, Linlin Du, Jun Chen, Xun Xu, Xiangui He, Jun Qiang

**Affiliations:** 1 Department of Preventative Ophthalmology, Shanghai Eye Disease Prevention and Treatment Center, Shanghai, China; 2 Department of Ophthalmology, Shanghai General Hospital, Shanghai Jiao Tong University School of Medicine, Shanghai, China

**Keywords:** Refractive error, Intermittent exotropia, Open-field autorefractor, Accommodative load

## Abstract

**Purpose:**

To investigate the difference of spherical equivalent (SE) and pupil diameter in adult patients with intermittent exotropia (IXT) under various viewing conditions before and after surgery.

**Methods:**

We retrospectively analyzed the medical records of 23 adult patients who underwent a surgery for IXT. The angle of deviation was measured by the prism and alternative cover test. Refractive error and pupil diameter were measured using the Grand Seiko WAM-5500 open-field autorefractor under binocular and monocular viewing conditions when patients stared at distance (6 m) and near (33 cm). Regression analyses were performed between accommodative load and the angle of deviation.

**Results:**

Twenty-three patients (10 males, 13 females) with a mean age of 31.17±8.95 years, of whom 13 (56.5%) had the right eye as the dominant eye. The mean angle of deviation at near and at distance was 69.57±26.37 and 65.43±28.92 prism diopters respectively. There were no significant differences in accommodative response and pupil diameter between the dominant and non-dominant eyes. SE decreased when patients changed from monocular to binocular viewing, and from distant to near viewing (all *P*< 0.05), so as the pupil diameter (all *P*< 0.001). During binocular, not monocular viewing, SE was significantly greater after operation than it was before operation (*P*< 0.001). Accommodative load and pupillary constriction narrowed (*p*< 0.001) after the operation. Linear regression analysis showed a correlation between the angle of deviation at distance and accommodative load at distance (r^2^=0.278, *p*=0.010) and at near (r^2^=0.332, *p*=0.005).

**Conclusion:**

In order to maintain ocular alignment, patients with IXT suffer a large accommodative load, which is related to the angle of deviation. Surgery helps eliminating extra accommodation.

## Background

Strabismus is an abnormal condition in which the visual axis of one eye deviates from parallel when the other eye is fixing [1]. Intermittent exotropia (IXT) is the most common type of divergent strabismus [2], and occurs frequently in Asian population [3]. In IXT, one eye intermittently deviates outward, especially when looking at the distance, in bright sunlight or when the patients is fatigued or distracted [4]. In the early stage of IXT, patients can maintain normal eye position and binocular visual function through the fusion and accommodative mechanism. With the progress of the disease, the fusion and accommodative convergence functions gradually weaken, resulting in exotropia getting more frequently, and sometimes developing into constant exotropia [5].

The severity of IXT mainly depends on the angle of deviation, the control ability of exodeviation and stereoacuity [1]. The main treatment of IXT is surgery to improve binocular function and stereoacuity, and to normalize the eye position [1]. The subjects of this study were patients with deviation degree at distance > 15 prism diopters (PD) who needed surgery. The preoperative and postoperative data were used to form self-control.

Accommodation, convergence, and pupillary constriction occur at the same time when the eye changes fixation from far to near. In the presence of IXT, due to insufficient fusional convergence, additional accommodative convergence is required to maintain binocular fusion [6, 7]. Most previous studies measured accommodative responses by using dynamic retinoscopy which is a subjective examination method, and the reliability of examination results depends on the examiner 's ability and experience [8].

In this study, an open-field autorefractor (WAM-5500; GrandSeiko, Fukuyama, Japan) was used to evaluate the objective refractive error and pupil diameter in patients with IXT. Due to the existence of internal fixation visual target, the ordinary computer optometry may induce instrument myopia caused by proximal accommodation, resulting in the deviation of measurement results [9, 10]. Compared with the traditional computer optometry, the Grand Seiko WAM-5500 open-field autorefractor overcomes the limitation of internal fixation visual target. It is widely used in clinic and research, such as the valid and repeatable measurement of pseudophakic eye refraction [11], and a screening method of over-refraction in the clinical fitting of multifocal contact lenses [12]. The open-field autorefractor can work under the conditions of open field of vision and binocular fixation, which is closer to the natural state of human eyes [13]. By adjusting the distance of fixation, the refractive error under different accommodation stimulus can be measured, and data of accommodation, convergence and pupillary constriction can be obtained.

At present, the research on the accommodative responses of patients with IXT is not sufficient, and only a few studies pay attention to the accommodation [14, 15]. No study has reported the SE before and after surgery in patients with IXT. And no study has examined the difference of SE and pupil diameter during monocular or binocular viewing conditions at near or distant fixation. Therefore, we performed this study to investigate the differences of SE before and after surgery and under different viewing conditions, which reveals the accommodative responses.

## Methods

### Study Population

This study included 23 adult patients who came to Shanghai Eye Diseases Prevention and Treatment Center and underwent a surgery for IXT by an ophthalmologist (Jun Qiang). Unilateral recession-resection surgery was conducted for patients with deviation degree ≤60 PD, and contralateral lateral rectus recession was added if the deviation degree was >60 PD. They were reexamined after operation. The reason for choosing adult patients was that adults complained about blurred vision and asthenopia more often than children, and they were better at keeping eye alignment during the open-field autorefractor exam.

Patients diagnosed with basic IXT according to the Burian classification [4] were included. The difference between their deviation degree at near and distance was less than 10 PD. In addition, they all had more than 15 PD of exodeviation at both distance and near fixation, whose best corrected visual acuity (BCVA) in either eye was 20/20 or better. Patients were excluded if they had amblyopia, anisometropia>2.00 D, vertical deviation >10 PD, A- or V-pattern strabismus, ocular motility disorders, other eye diseases that could affect vision, and a history of ophthalmic surgery. The study was approved by the Institutional Ethics Committee of Shanghai General Hospital, Shanghai Jiao Tong University School of Medicine (2021KY093) and adhered to the tenets of the Declaration of Helsinki. In this study, only the clinical data of patients were collected, and the treatment was not intervened. The data are anonymous, and the requirement for informed consent was therefore abandoned, which was also approved by the Institutional Ethics Committee of Shanghai General Hospital, Shanghai Jiao Tong University School of Medicine.

### Ophthalmologic examination

All patients underwent a comprehensive ophthalmologic examination performed by a veteran ophthalmologist (Jun Qiang). The prism and alternative cover test (PACT) [16, 17] were performed at least three times until stable data was obtained for measuring the angle of deviation at near (33 cm) and distance (6 m) both before and after the operation. During the examination, patients wore corrective glasses to achieve BCVA of at least 20/20 in both eyes. Ordinary refractive error was measured by an autorefractor (RM8800; Topcon, Tokyo, Japan) without cycloplegia. Each eye was measured at least three times, and then the average value was taken. If any two measurements changed by more than 0.50 D, the readings were discarded and the eye was remeasured. The SE was calculated by the standard formula of the sum of the sphere and half of the cylinder. Ocular dominance was determined based on the hole-in-a-card test [18] (three times), and the eye that can see the distant target was considered as the dominant eye.

Refractive error that reflects the accommodative response was measured by an experienced optometrist to confirm maintenance of ocular alignment during measurements. Both eyes were measured using the Open-field autorefractor (WAM-5500; GrandSeiko, Fukuyama, Japan) without corrective glasses. This machine was used to measure refractive error (presented as SE) under binocular and monocular viewing conditions when patients stared at distance (6 m) and near (33 cm). The pupil diameter was measured by an eye-gaze tracker on the Open-field autorefractor [19]. Patients were asked to stare at the center of a Maltese cross on a white card. In each condition, at least five measurements were required. During the test, the lighting intensity of the room was constant. Binocular viewing with the ocular alignment was assessed first to avoid the interruption of fusion, and then monocular viewing was evaluated. The left and right eyes were examined in random order by occluding the opposite eye. Accommodative load was calculated as the difference of SE between binocular and monocular viewing conditions [7].

### Statistical analysis

All analyses were performed using SPSS 25.0 (SPSS Inc., Chicago, Illinois, USA). Continuous variables accorded with normal distribution such as age, deviation degree and refractive error (presented as SE) were expressed as mean ± standard deviation. Meanwhile, continuous variables that did not accord with normal distribution were expressed as medium (min, max). Counting data such as gender and ocular dominance were described by frequency (%). The paired-samples t test and Wilcoxon singed rank test were used to compare the accommodative response, pupil diameter and deviation degree under different conditions. Linear regression analyses were employed to assess the relationship between accommodative load and deviation degree. A two-sided *p* < 0.05 was considered statistically significant.

### Results

A total of 23 patients aged 18 to 60 years (10 males, 13 females) were included in this study. The median (min, max) postoperative follow-up time was 43 (26, 271) days. Table 1 displayed the basic characteristics of patients with basic IXT. The mean age of all patients was 31.17±8.95 years (range from 18 to 57). 56.5% (13/23) of the patients’ dominant eye was the right eye, and 43.5% (10/23) of the patients’ dominant eye was the left eye. The mean (min, max) angle of deviation at near and at distance was 69.57±26.37 (30, 140) PD and 65.43±28.92 (20, 140) PD, respectively. There was no significant difference in characteristics between male and female (Table 1).

**Table 1:** Characteristics of patients with basic intermittent exotropia^a^

	Total (***N***=23)	Male (***N***=10)	Female (***N***=13)	***P*** value
Age, year	31.17±8.95	30.60±6.85	31.62±10.55	0.794*
Ocular dominance, No. (%)				
Right	13 (56.5)	6 (60.0)	7 (53.8)	0.768‡
Left	10 (43.5)	4 (40.0)	6 (46.2)	
Deviation degree at near, PD	69.57±26.37	76.50±36.82	64.23±13.67	0.338*
Deviation degree at distance, PD	65.43±28.92	71.00±40.47	61.15±16.09	0.482*
Ordinary refractive error, D^b^	-3.10±1.98	-2.86±1.72	-3.30±2.23	0.616*

*PD* prism diopter, *D* diopter

^a^Data are presented as mean ± standard deviation unless otherwise indicated

^b^Ordinary refractive error was measured by the RM8800 autorefractor. Right eyes were chosen for data analysis since the SE of the right and left eyes were highly correlated

*Independent-samples t test ‡ Chi-square test

### SE and pupil diameter in different viewing conditions

Since there were no significant differences in accommodative response and pupil diameter between the dominant and non-dominant eyes under various circumstances, whether monocular or binocular, preoperative or postoperative, distant or near viewing conditions, the data of the dominant eye were used in subsequent analysis (Table 2).

**Table 2:** SE and pupil diameter of dominant eye and non-dominant eye in different status (*N*=23)

	Spherical equivalent, D			Pupil diameter, mm		
	Dominant eyes	Non-dominant eyes	***P*** value	Dominant eyes	Non-dominant eyes	***P*** value
**Preoperative distant vision**						
** Binocular**^**a**^	-3.13 (-8.25, 0.63)	-3.50 (-8.58, 0.63)	0.175*	4.51±1.36	4.49±1.40	0.795†
** Monocular**	-1.76±2.29	-1.89±1.95	0.532†	5.96±0.93	6.07±0.79	0.110†
**Preoperative near vision**						
** Binocular**	-4.01±2.55	-3.91±2.05	0.690†	3.90±1.29	4.02±1.38	0.221†
** Monocular**	-2.32±1.79	-2.39±1.74	0.590†	5.71±1.06	5.85±1.02	0.176†
**Postoperative distant vision**						
** Binocular** ^**a**^	-1.38 (-5.83, 0.88)	-1.75 (-6.50, 0.88)	0.385*	5.67±1.16	5.67±1.08	0.910†
** Monocular** ^**a**^	-1.25 (-5.13, 1.00)	-1.38 (-5.63, 1.13)	0.097*	6.04±1.19	6.18±1.03	0.130†
**Postoperative near vision**						
** Binocular**	-2.82±1.79	-3.08±1.64	0.259†	5.24±1.35	5.26±1.31	0.785†
** Monocular**	-2.53±1.46	-2.61±1.54	0.534†	5.87±1.08	5.79±1.20	0.453†

^a^ Data are presented as medium (min, max)

* Wilcoxon signed-rank test † Paired-samples t test

Refractive error and pupil diameter of dominant eye measured by the open-field autorefractor in different status were shown in Table 3. Before the operation, the SE of dominant eye at distance and near was both greater during monocular viewing condition compared with that during binocular viewing condition (both *P*<0.001). The same result appeared after the operation. Although the SE of dominant eye at distance and near was slightly greater during monocular viewing condition than that during binocular viewing condition, the difference of SE between monocular and binocular viewing was significant (*P*=0.023, 0.030, respectively). Further, the SE of dominant eye in all cases significantly decreased when the patients with IXT stared from distant to near (all *P*<0.01). IXT patients’ pupil diameter under monocular viewing condition was significantly larger than it was under binocular viewing condition in all situations (all *P*<0.001). However, when the patients looked closer, although the pupil diameter showed a narrowing trend, the difference was not significant in one case, that was monocular viewing after operation (*p*=0.163).

**Table 3:** SE and pupil diameter of dominant eye in different status (*N*=23)

	Spherical equivalent, D ^a^			Pupil diameter, mm		
	Binocular	Monocular	***P*** value	Binocular	Monocular	***P*** value
**Preoperative**						
** Distant**	-3.13 (-8.25, 0.63)	-1.13 (-5.63, 1.75)	<0.001*	4.51±1.36	5.96±0.93	< 0.001†
** Near**	-4.00 (-8.25, 0.25)	-1.63 (-5.75, 0.88)	<0.001*	3.90±1.29	5.71±1.06	< 0.001†
*** P*** **value**	0.003*	0.014*		< 0.001†	0.014	
**Postoperative**						
** Distant**	-1.38 (-5.38, 0.88)	-1.25 (-5.13, 1.00)	0.023*	5.67±1.16	6.04±1.19	< 0.001†
** Near**	-2.38 (-6.00, 0.38)	-2.16 (-5.63, 0.00)	0.030*	5.24±1.35	5.87±1.08	< 0.001†
*** P*** **value**	0.001*	0.008*		0.001†	0.163†	

^a^ Data are presented as medium (min, max)

* Wilcoxon signed-rank test † Paired-samples t test

### Changes of deviation degree and SE after operation

**Fig 1:**
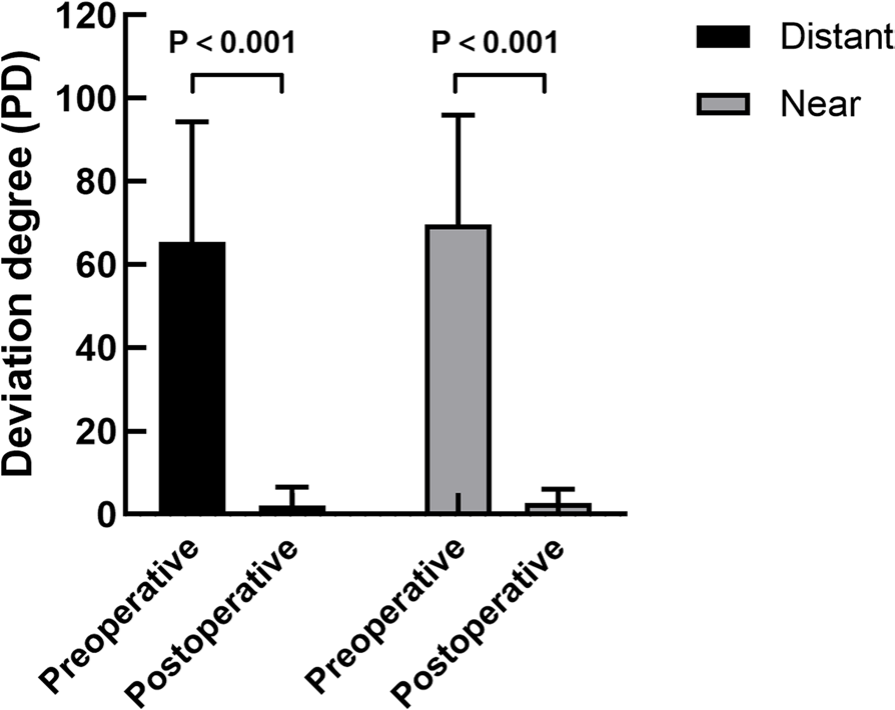
Variation of deviation degree after operation (*N*=23). The deviation degree at distance was shown by black boxes and the deviation degree at near was shown by grey boxes. Data are presented as mean ± SD. *P*<0.001 by Paired-samples t test

Figure 1 and Table 4 presented the impact of the operation on patients with basic IXT. Operated patients showed a significant decrease in the angle of deviation at distance and near (both *P*<0.001; Fig 1). During monocular viewing, there was no significant difference between preoperative and postoperative SE of dominant eye at distance and near (*p*=0.626, 0.188, respectively; Table 4). However, during binocular viewing, SE of dominant eye was significantly greater after operation than it was before operation during distant and near viewing conditions (both *P*<0.001). Table 4 also compared the accommodative load of dominant eye in IXT patients before and after operation. During distant viewing condition, the difference in SE between binocular and monocular viewing conditions for dominant eye significantly narrowed after the operation (*p*< 0.001). Similarly, during near viewing condition, the amount of postoperative accommodative load of dominant eye was significantly smaller than that before operation (*p*< 0.001). In addition, pupillary constriction at distant and near fixation showed a significant decreasing trend after operation (both *P*<0.001; Table 4).

**Table 4:** Accommodative response of dominant eye before and after operation (*N*=23)

	Preoperative	Postoperative	***P*** value
**Distant spherical equivalent, D**			
** Binocular**	-3.13 (-8.25, 0.63)	-1.38 (-5.83, 0.88)	< 0.001*
** Monocular**	-1.13 (-5.63, 1.75)	-1.25 (-5.13, 1.00)	0.626*
**Near spherical equivalent, D**			
** Binocular**	-4.01±2.55	-2.82±1.79	0.001†
** Monocular**	-2.32±1.79	-2.53±1.46	0.188†
**Accommodative load** ^**a**^**, D**			
** Distant**	-1.00 (-5.00, 0.38)	-0.13 (-0.75, 0.50)	< 0.001*
** Near**	-1.69±1.16	-0.29±0.64	< 0.001†
**Pupillary constriction** ^**b**^**, mm**			
** Distant**	1.41±0.86	0.31±0.38	< 0.001†
** Near**	1.74±0.75	0.61±0.64	< 0.001†

^a^ Accommodative load refers to the difference in SE of dominant eye between binocular and monocular vision

^b^ Pupillary constriction refers to the difference in pupil diameter of dominant eye between monocular and binocular vision

* Wilcoxon signed-rank test † Paired-samples t test

### Relationship between the accommodative load and the deviation degree

**Fig 2:**
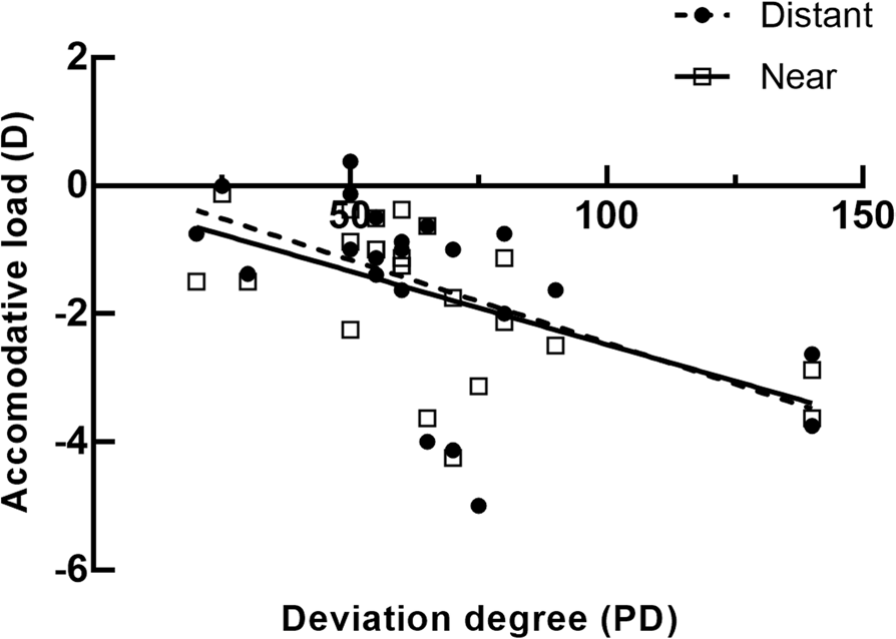
Scatter plot showing the regression between the accommodative load and the deviation degree at distance. The accommodative load of dominant eye at 6m was shown by dotted line (r^2^=0.278, *p*=0.010). The accommodative load of dominant eye at 33cm was shown by solid line r^2^=0.332, *p*=0.005)

Linear regression analysis was performed to evaluate the correlation between the accommodative load of dominant eye and the angle of deviation. The angle of deviation at distance correlated significantly with accommodative load of dominant eye at distance (6m) (r^2^=0.278, *p*=0.010; Fig 2) and at near (33cm) (r^2^=0.332, *p*=0.005; Fig 2). Whether patients with IXT stared at distance or at near, the difference in SE between binocular and monocular vision for dominant eye decreased with the increase of the angle of exodeviation at distance, suggesting that the more serious IXT was, the greater the accommodative load was.

## Discussion

Our study measured the SE and pupil diameter of each eye during monocular or binocular viewing conditions at near or distant fixation before and after surgery. We also investigated the relationship between the accommodative load and the angle of deviation at distance. Previous studies on the accommodative response were mostly carried out by dynamic retinoscopy [8, 20]. However, retinoscopy is limited by the time required, the patient's discomfort and the examiner's subjective judgment. Therefore, the application of open-field autorefractor is not only more applicable, but also provides more reliable data.

Our study showed that there was no significant difference in SE measured by the Grand Seiko WAM-5500 between dominant and non-dominant eyes in patients with basic IXT, which was contrary to the results of others [5, 21]. Their results found that the accommodative responses between dominant and non-dominant eye in patients with IXT was not completely consistent and balanced, and this imbalance was likely caused by the competition between two eyes. The possible reason was that the SE of basic IXT patients in our study was measured without corrective glasses, which is different from the accommodative response directly measured with corrective glasses through the Grand Seiko WAM-5500. The reason why we chose not to let patients wear corrective glasses to achieve BCVA ≥ 20 / 20 is that our purpose is to compare the difference of refractive error under various viewing conditions, rather than the absolute difference of accommodative response.

Most studies used healthy normal people as controls for group analysis, while this study recruited patients with corrected strabismus after surgery as their own control to minimize interindividual variability. We found that the SE of patients with basic IXT after operation was significantly higher than that before operation only in the state of binocular fixation, which was basically consistent with previous studies using normal people as controls [7].

Fusional convergence, accommodative convergence, and tonic convergence are the three innervations [22]. Patients with IXT utilize mechanism of fusion and accommodative convergence to maintain eye alignment. The accommodative convergence will increase to maintain the fusion image if the fusion convergence mechanism is insufficient to maintain normal function [23]. Some studied reported myopic patients with IXT may experience faster myopia progression than healthy peers without IXT [24, 25]. It is as expected that the SE of dominant eye during binocular fixation was lower than that during monocular fixation in our study, suggesting that patients with IXT need more accommodative convergence to maintain binocular fusion vision. The same situation occurred in the process from distance to near fixation. We found that the SE of dominant eye significantly decreased when focusing on a near target, which supports the idea that patients with IXT require excessive accommodation to obtain a single binocular fusion vision when they stare close. In brief, patients with basic IXT bear lower SE of dominant eye during binocular viewing of a near target, which may lead to myopia progression.

Since accommodation, convergence, and pupillary constriction are closely related triad, pupil constriction during binocular near viewing conditions were observed naturally in our study. However, no evidence for the direct relationship between pupil diameter and accommodative responses was found in previous studies [26, 27]. And in our study (data not shown), pupil constriction was also not significantly related with the angle of deviation and accommodative load.

We also found the accommodative load (referring to the difference in SE between binocular and monocular viewing conditions) of dominant eye significantly narrowed after the operation, which provided further evidence for the requirement of excessive accommodation in patients with basic IXT to maintain binocular fusion vision. Moreover, with the increase of exodeviation degree, that is, the aggravation of IXT, greater accommodative load is needed both under distant and near vision. A study by Ahn et al. found the accommodative response increased as the distant exodeviation degree increased in patients with IXT [6]. The significant correlation between the accommodative response and the size of exodeviation degree also backs up the idea that the angle of deviation affects the accommodation required to maintain ocular alignment.

We speculate that the decrease of SE under binocular viewing condition leads to the blurred vision and asthenopia of adult patients with IXT. However, in clinical work, the ophthalmologist (Jun Qiang) has found that few children complain about blurred vision and asthenopia. Meanwhile, studies reported that diplopia was the most common symptom in adults, while it seldom occurred in childhood [28, 29], indicating that the accommodative or tonic convergence was more active in children than in adults [28]. Furthermore, Yang et al. found that IXT children with a dominant eye had an asymmetrical accommodative response between the two eyes during binocular viewing [21]. At the next stage, we may consider conducting children's research to explore the difference of accommodative load between children and adults.

This study has obvious limitations. Firstly, all data of refractive errors were obtained without cycloplegic refraction, which is common in studies where subjects were adults [30, 31]. Secondly, as accommodative convergence/accommodation (AC/A) is informative for patients with convergence insufficiency and true divergence excess type IXT, while it is within the normal range for basic IXT, we did not measure the value of AC/A. Thirdly, the small sample size of this study and the inclusion of only basic type of IXT patients may limit the extrapolation of results. In the future, more different subtypes of IXT patients can be investigated to obtain and analyze their SE and pupil size.

## Conclusions

Our study found SE and pupil diameter changed in patients with basic IXT from preoperative to postoperative, from binocular viewing to monocular viewing, and from distant fixation to near fixation, supporting the idea that accommodative convergence is a mechanism to maintain ocular alignment.

## Data Availability

The datasets used and/or analyzed during the current study are available from the corresponding author on reasonable request.

## Abbreviations

SE: Spherical equivalent
IXT: Intermittent exotropia
BCVA: Best corrected visual acuity
PD: Prism diopter
D: diopter
PACT: Prism and alternative cover test
